# Association between serum lactate levels and mortality in patients with cardiogenic shock receiving mechanical circulatory support: a multicenter retrospective cohort study

**DOI:** 10.1186/s12872-020-01785-7

**Published:** 2020-11-24

**Authors:** Fernando Luís Scolari, Daniel Schneider, Débora Vacaro Fogazzi, Miguel Gus, Marciane Maria Rover, Marcely Gimenes Bonatto, Gustavo Neves de Araújo, André Zimerman, Daniel Sganzerla, Lívia Adams Goldraich, Cassiano Teixeira, Gilberto Friedman, Carisi Anne Polanczyk, Luis Eduardo Rohde, Regis Goulart Rosa, Rodrigo Vugman Wainstein

**Affiliations:** 1 Research Projects Office, Hospital Moinhos de Vento (HMV), Rua Ramiro Barcelos 630, 10º andar, Porto Alegre, RS 90035-001 Brazil; 2 Division of Cardiology, Hospital de Clínicas de Porto Alegre (HCPA), Rua Ramiro Barcelos 630, Porto Alegre, RS 90035-001 Brazil; 3 Division of Cardiology, HMV, Rua Tiradentes, 333, Porto Alegre, RS 90560-030 Brazil; 4 Heart Failure and Transplant Division, Instituto de Cardiologia – Fundação Universitária de Cardiologia, Av. Princesa Isabel, 395, Porto Alegre, RS 90040-371 Brazil; 5 Cardiology Department, Transplant Division, Irmandade Hospital da Santa Casa de Misericórdia de Curitiba, Praça Rui Barbosa, 694, Curitiba, PR 80010-030 Brazil; 6 Postgraduate Program in Health Sciences: Cardiology and Cardiovascular Sciences,, UFGRS, Rua Ramiro Barcelos, 2350, Porto Alegre, RS 90035-007 Brazil; 7 Division of Cardiology, London Health Sciences Center and Western University, London, Canada; 8 Heart Transplant and Mechanical Circulatory Support Program, Division of Cardiology, HCPA, Rua Ramiro Barcelos, 2350, Porto Alegre, RS 90035-007 Brazil; 9 Division of Critical Care Medicine, HMV, R. Tiradentes, 333, Porto Alegre, 90560-030 Brazil; 10 Division of Critical Care Medicine, HCPA, Rua Ramiro Barcelos 630, Porto Alegre, 90035-001 Brazil; 11 Universidade Federal do Rio Grande do Sul, R. Tiradentes, 333, Porto Alegre, 90560-030 Brazil

**Keywords:** Cardiogenic shock, Extracorporeal membrane oxygenation, Impella, Mechanical circulatory support, Lactic acid

## Abstract

**Background:**

To evaluate the prognostic value of peak serum lactate and lactate clearance at several time points in cardiogenic shock treated with temporary mechanical circulatory support (MCS) using veno-arterial extracorporeal membrane oxygenation (VA-ECMO) or Impella CP^®^.

**Methods:**

Serum lactate and clearance were measured before MCS and at 1 h, 6 h, 12 h, and 24 h post-MCS in 43 patients at four tertiary-care centers in Southern Brazil. Prognostic value was assessed by univariable and multivariable analysis and receiver operating characteristic (ROC) curves for 30-day mortality.

**Results:**

VA-ECMO was the most common MCS modality (58%). Serum lactate levels at all time points and lactate clearance after 6 h were associated with mortality on unadjusted and adjusted analyses. Lactate levels were higher in non-survivors at 6 h, 12 h, and 24 h after MCS. Serum lactate > 1.55 mmol/L at 24 h was the best single prognostic marker of 30-day mortality [area under the ROC curve = 0.81 (0.67–0.94); positive predictive value = 86%). Failure to improve serum lactate after 24 h was associated with 100% mortality.

**Conclusions:**

Serum lactate was an important prognostic biomarker in cardiogenic shock treated with temporary MCS. Serum lactate and lactate clearance at 24 h were the strongest independent predictors of short-term survival.

## Background

Cardiogenic shock is a high-mortality condition with increasing incidence [1]. The hemodynamic impairment caused by cardiogenic shock triggers an inflammatory cascade, which leads to circulatory collapse and tissue perfusion impairment [2]. Lactate is a metabolic byproduct of anaerobic glycolysis and a reliable marker of tissue hypoperfusion [3]. It has been used both as prognostic variable and as therapeutic target in different clinical scenarios of shock [4, 5]. Treating cardiogenic shock with MCS may enhance macro- and microcirculation, improving tissue perfusion [6].The increase in tissue oxygenation might reduce lactate production, which makes it a good biomarker [7–9].

The judicious selection of patients eligible for MCS therapy is essential, since it increases cost exponentially and may be futile in advanced shock status [10]. Several risk scores have been proposed to establish prognosis and help in MCS selection in cardiogenic shock. However, these scores are known to be inaccurate and too complex to be used at bedside [11], especially in patients receiving MCS [12]. In this sense, arterial lactate is a widely available biomarker that can be used at bedside to evaluate prognosis [13]. Although any single lactate measurement might have prognostic value in patients with cardiogenic shock, it is unclear whether peak serum lactate, lactate clearance, or measurements obtained at any one time point post-MCS have the greatest prognostic predictive accuracy [5, 8, 14]. Recently, a large cohort of patients submitted to intra-aortic balloon pump (IABP) as treatment of cardiogenic shock evaluated the prognostic role of arterial lactate in several time-points, showing that its measure after 8 h was superior to baseline and clearance values [13]. However, IABP provides a lower increase in cardiac output in comparison to other MCS such as Impella CP^®^ or veno-arterial extracorporeal membrane oxygenation (VA-ECMO) [6]. Previous studies on the role of arterial lactate in cardiogenic shock with MCS are heterogeneous in study population and device selected [8, 14, 15]. Moreover, the ideal cutoff lactate value to identify patients with better prognosis is yet to be established in this scenario. Within this context, we conducted an observational study to evaluate the prognostic role of serum lactate measurements and lactate clearance over time in patients with several cardiogenic shock etiologies treated with temporary MCS.

## Methods

### Study population and design

This is a retrospective analysis of a cohort of cardiogenic shock patients treated with Impella CP^®^ or VA-ECMO between April 2017 and July 2019 at four tertiary centers in Southern Brazil. All centers were participants of the *Qualificação do uso de Dispositivos de Assistência Circulatória no SUS* (Qualification for use of MCS devices in the Brazilian Unified Health System) project*.* Center selection criteria included tertiary centers with a catheterization laboratory able to provide 24-h support and intensive care unit with a dedicated team for patients on temporary MCS. Eligibility criteria included patients in cardiogenic shock treated with MCS for whom complete clinical records, serum lactate levels measured at pre-established time points, and data at 30-day follow-up were available. The exclusion criteria were patients in whom MCS was used to support high-risk percutaneous coronary intervention, and all settings other than cardiogenic shock. Patients were followed for a 30-day period after MCS device placement. The project was approved by the ethics committees of all participating centers and complied with the principles of the Helsinki Declaration (2008 revision). Written informed consent was obtained from all patients or, if the patient was in no clinical condition to provide consent, from a legal guardian or next of kin.

### Indications and management of MCS

Temporary MCS was indicated for patients with cardiogenic shock and persistent hemodynamic instability despite initial management with vasopressors and/or revascularization when needed. Hemodynamic instability was defined as a systolic blood pressure ≤ 90 mmHg despite inotrope/vasopressor support, signs of end-organ failure (clammy skin, capillary filling time > 3 s, urine output < 0.5 mL/kg/h, lactate level > 4 mmol/L), and low cardiac output (< 2.2 L/min/m^2^ if receiving inotropes/vasopressors or < 1.8 L/min/m^2^ without inotropes/vasopressors).

Impella CP^®^ (Abiomed Europe GmbH, Aachen, Germany) and VA-ECMO (MAQUET Holding B.V.& Co. KG, Rastatt, Germany) were available at all centers. Among patients with clinical indications for MCS, the etiologies of cardiogenic shock included acute myocardial infarction, acute decompensated heart failure, postcardiotomy shock, primary allograft dysfunction after heart transplantation, and myocarditis. Selection of the MCS device and overall patient management were left at the discretion of each center, according to clinical indication and center experience. All participating centers were trained in MCS indication and management according to current guidelines and ELSO (Extracorporeal Life Support Organization) [16].

### Lactate measurement

All serum lactate levels were measured in arterial blood gas samples. Baseline lactate was defined as the last lactate level measured before device implantation. Any levels prior to that were not considered for analysis. Lactate levels after MCS were measured at predetermined time points (1 h, 6 h, 12 h, and 24 h after device implantation). For imputation of missing lactate values (10%), the mean between the preceding and subsequent available time points was used. Lactate clearance was determined by the following formula: [(lactate at time point of interest − initial lactate)/initial lactate*100] [8].

### Follow-up and outcomes

All patients were followed after MCS weaning up to hospital discharge. If it occurred before 30 days of follow-up, patients were considered alive if reached by telephone or had a medical record within 30 days. The primary outcome was defined as all-cause death. Survivors were defined as patients alive after 30 days weaned from MCS.

### Statistical analysis

All continuous variables were tested for normality by the Shapiro–Wilk test and histogram analysis. If the assumption of normality was rejected, data were reported as median [interquartile range (IQR)]. Categorical variables were expressed as number and percentage. A Mann–Whitney test was used to compare lactate levels before and after MCS device implantation. The chi-square test was used to compare categorical variables. For evaluation of prognostic value, the probability of death during follow-up was estimated for initial, 1 h, 6 h, 12 h, 24 h and overall effect for lactate (in mmol/L) and lactate clearance (in %), with the use of univariable and multivariable logistic regression models. For the multivariable analysis, three logistic regression models were created with the following adjustment variables: (1) device type, pre-device cardiac arrest, and center; (2) age, device type, shock to support time, and center; (3) Simplified Acute Physiology Score III (SAPS3) and Sequential Organ Failure Assessment Score (SOFA) and center. We performed a sensitivity analysis for lactate levels and lactate clearance missing inputs. All variables were tested for multicollinearity in the multivariable models by the variance inflation test. The receiver operating characteristic (ROC) curve was used to evaluate the ability of different lactate levels to predict 30-day mortality. To identify lactate cutoff points, Youden’s J index was calculated as: J = sensitivity + (specificity − 1). Sensitivity (S) and positive predictive value (PPV) were calculated for the time points of interest. Time to event analysis was calculated with Kaplan–Meier curves for each cutoff of lactate level or lactate clearance. Patients were censored at death or 30 days. Significance was accepted at *P* < 0.05 for all tests. Data were analyzed in SPSS, Version 20.0 for Windows (SPSS Inc., Chicago, IL, USA), and R software (R Foundation for Statistical Computing, Vienna, Austria; http://www.R-project.org).

## Results

The cohort comprised 48 patients, of whom 5 were excluded because the Impella CP^®^ was used during high-risk percutaneous coronary intervention. Baseline characteristics and clinical data of the 43 patients included in the analysis are summarized in Table 1. Patients enrolled at each center, their CS etiology and device employed are shown at Additional file 1: Table S1. The median (IQR) age was 57.0 (42.0–63.0) years; 33 (77%) were male. Analysis of comorbidities showed history of hypertension in 19 (44%), diabetes in 15 (35%), and glomerular filtration rate < 60 mL/min/1.73m^2^ in 27 patients (63%). The most common etiology of cardiogenic shock was acute myocardial infarction, in 19 patients (44%), followed by acute decompensation of chronic heart failure in 10 (24%), primary graft failure in 4 (9%), cardiac arrest in 4 (9%), postcardiotomy shock in 3 (7%), pulmonary thromboembolism in 2 (5%), and myocarditis in 1 (2%). VA-ECMO was used in 25 patients (58%), the Impella CP^®^ in 13 (30%), and combined VA-ECMO with Impella CP^®^ in 5 (12%). Median time from shock team consult to MCS deployment was 3.0 (1.0–16.5) hours, and the median duration of support was 2.0 (1.0–5.0) days. At MCS initiation, 33 patients (77%) were receiving norepinephrine, at a median dose of 0.5 (0.25–0.64) mcg.kg.min^−1^; the most commonly used inotrope was dobutamine (37% of patients), and its median dose was 5.2 (4.5–8.0) mcg.kg.min^−1^. The median initial serum lactate level was 6.1 mmol/L (2.8–11.5 mmol/L), and the central venous oxygen saturation was 68.0% (54.0–74.6%).

**Table 1:** Demographic and clinical data of 43 patients with cardiogenic shock who received mechanical circulatory support with veno-arterial extracorporeal membrane oxygenation (VA-ECMO) or an Impella CP^®^ device

	All (*n* = 43)	Survivors (*n* = 12)	Non-survivors (*n* = 31)
Clinical and demographic data			
Age, years, median (IQR)	57.0 (43.0 to 63.0)	57.0 (42.5 to 61.5)	59.0 (46.0 to 64.0)
Male sex (%)	33 (76.7)	9 (75.0)	24 (77.4)
Hypertension (%)	29 (67.4)	5 (41.6)	24 (77.4)
Diabetes mellitus (%)	15 (34.8)	1 (8.3)	14 (45.1)
COPD (%)	3 (6.9)	0 (0)	3 (9.6)
Pre-device cardiac arrest (%)	14 (32.5)	6 (50)	8 (25.8)
Etiology of cardiogenic shock			
Acute myocardial infarction (%)	19 (44.1)	6 (50)	13 (41.9)
Acute decompensation of chronic heart failure (*%*)	10 (23.2)	2 (16.6)	8 (25.8)
Primary transplant graft failure (%)	4 (9.3)	2 (16.6)	2 (6.4)
Cardiac arrest (%)	4 (9.3)	1 (8.3)	3 (9.6)
Postcardiotomy syndrome (%)	3 (6.9)	0 (0)	3 (9.6)
Pulmonary thromboembolism (%)	2 (4.6)	0 (0)	2 (6.4)
Myocarditis (%)	1 (2.3)	1 (8.3)	0 (0)
Device characteristics			
VA-ECMO (%)	25 (58.1)	6 (50.0)	19 (61.2)
Impella CP^®^ (%)	13 (30.2)	5 (41.6)	8 (25.8)
VA-ECMO + Impella^®^ (%)	5 (11.6)	1 (8.3)	4 (12.9)
Shock to support time, hours, median (IQR)	3.0 (1.0 to 16.5)	3.5 (2.25 to 20.5)	2.5 (1.0 to 16.5)
Time on support, days. median (IQR)	2.0 (1.0 to 5.0)	2.0 (1.25 to 3.0)	2.0 (1.0 to 6.0)
Critical illness variables			
Arterial lactate, mmol/L, median (IQR)	6.1 (2.8 to 11.5)	4.0 (2.62 to 6.3)	7.5 (2.8 to 12.0)
Serum creatinine, mg/dL, median (IQR)	1.5 (1.1 to 1.8)	1.4 (0.8 to 1.8)	1.5 (1.2 to 1.8)
Arterial pH, median (IQR)	7.29 (7.23 to 7.34)	7.28 (7.23 to 7.34)	7.31 (7.22 to 7.37)
Arterial HCO_3,_ mEq/L, median (IQR)	18.7 (15.0 to 22.0)	17.6 (14.0 to 22.1)	18.8 (15.3 to 22.4)
Central venous oxygen saturation, %, median (IQR)	68.0 (54.0 to 74.6)	68.0 (54.0 to 83.1)	64.4 (56.9 to 72.9)
SAVE score, median (IQR)^a^	− 9.0 (− 11.0 to 0)	− 8.9 (− 10.0 to 3.0)	− 9.0 (− 11.0 to − 1.2)
ENCOURAGE score, median (IQR)^b^	23.5 (18.5 to 28.0)	24.0 (12.0 to 26.0)	23.0 (19.0 to 28.0)
SAPS 3 score, median (IQR)^c^	70.0 (60.0 to 84.0)	61.0 (58.2 to 70.0)	72.0 (67.0 to 88.0)
SOFA score, median (IQR)^d^	13.0 (10.0 to 15.0)	11.5 (10.0 to 14.7)	13.0 (10.0 to 15.0)

COPD, chronic obstructive pulmonary disease; ECMO, extracorporeal membrane oxygenation; ENCOURAGE, prediction of cardiogenic shock outcome for acute myocardial infarction patients salvaged by VA-ECMO; IQR, interquartile range (p25–p75); MCS, mechanical circulatory support; SAPS 3, Simplified Acute Physiology Score III; SAVE, Survival After Veno-arterial ECMO score; SOFA, Sequential Organ Failure Assessment score; VA, veno-arterial

^a^Score ranges from -15 to 15, with higher scores indicating lower mortality. For example, a score of -9 is associated with 70% mortality [17]

^b^Score ranges from 0 to 28, with higher scores indicating higher mortality. For example, a score of 23.5 is associated with 76% mortality at 30 days [18]

^c^Score ranges from 0 to 207, with higher scores indicating higher mortality. For example, a score of 70 is associated with 55% in-hospital mortality [19]

^d^Score ranges from 6 to 24, with higher scores indicating higher mortality. For example, a score of 13 is associated with 85% mortality [20]

At 30-day follow-up, 24 (56%) patients died on MCS, 19 (44%) were weaned from MCS, and 12 (28%) were discharged from hospital and were alive at 30-day follow up. Risk scores for patients treated with MCS were calculated at admission. Specific scores for cardiogenic shock treated with MCS (such as SAVE score and ENCOURAGE score) and a general intensive care unit (ICU) score (SOFA) did not differ between groups and were not associated with 30-day mortality in the logistic regression models. However, SAPS 3, a general ICU score, differed between survivors and non-survivors (61.0 [58.2–70.0] vs. 72.0 [67.0–88.0], *P* = 0.01) and predicted 30-day mortality in unadjusted analysis (OR = 1.01; CI 1.00–1.02, *P* = 0.002). The predicted mortality calculated through these risk scores ranged from 55 to 85% [17–20].

### Lactate levels and lactate clearance as predictors of mortality

Lactate levels were associated with different odds ratios (ORs) of mortality across time points, with a progressive increase in OR over time and the greatest magnitude at 24 h in the unadjusted model with similar trends in the adjusted models. The OR for overall effect of lactate ranged from 1.26 to 1.73 across adjusted models. Lactate clearance was significantly associated with mortality only after 6 h, in both unadjusted and adjusted models (Table 2). The unadjusted analysis of the variables included in the models are shown in Additional file 1: Table S2. Sensitive analyses of serum lactate and lactate clearance for missing inputs showed similar results (Additional file 1: Table S3), as well as the sensitive analysis for cardiogenic shock etiology (Additional file 1: Table S4).

**Table 2:** Association of serum lactate levels and clearance with 30-day mortality in unadjusted and adjusted analyses

	Unadjusted			Adjusted (model 1)^a,d^			Adjusted (model 2)^b,d^			Adjusted (model 3)^c,d^		
	OR	95% CI	*P*	OR	95% CI	*P*	OR	95% CI	*P*	OR	95% CI	*P*
Lactate, baseline	1.15	1.00–1.39	0.04	1.13	0.98–1.37	0.09	1.19	1.01–1.48	0.03	1.21	1.01–1.55	0.03
Lactate, 1 h	1.13	1.00–1.33	0.05	1.11	0.97–1.32	0.14	1.21	1.02–1.50	0.02	1.29	1.07–1.73	0.005
Lactate, 6 h	1.18	1.03–1.47	0.01	1.16	1.01–1.45	0.03	1.27	1.07–1.64	0.004	1.34	1.10–1.95	0.01
Lactate, 12 h	1.27	1.05–1.76	0.005	1.25	1.04–1.74	0.01	1.46	1.13–2.22	0.001	1.62	1.19–3.03	< 0.001
Lactate, 24 h	1.76	1.13–4.33	0.001	1.63	1.11–3.88	0.001	2.37	1.24–6.90	< 0.001	5.86	1.53–67.86	< 0.001
Overall lactate^e^	1.27	1.06–1.70	0.03	1.26	1.04–1.71	0.01	1.54	1.16–2.50	< 0.001	1.73	1.20–3.48	< 0.001
Clearance, 1h^f,g^	1.00	0.98–1.01	0.60	1.00	0.98–1.01	0.81	0.99	0.97–1.01	0.31	0.99	0.97–1.01	0.10
Clearance, 6h^f,g^	0.98	0.97–0.99	0.01	0.98	0.97–0.99	0.01	0.97	0.95–0.99	0.002	0.97	0.94–0.99	0.002
Clearance, 12h^f,g^	0.98	0.96–0.99	0.006	0.98	0.96–0.99	0.005	0.97	0.94–0.99	< 0.001	0.97	0.93–0.99	< 0.001
Clearance, 24h^f,g^	0.95	0.91–0.98	< 0.001	0.95	0.90–0.99	< 0.001	0.94	0.87–0.98	< 0.001	0.97	0.78–0.98	< 0.001

CI, confidence interval; OR, odds ratio

^a^Model 1: adjusted by type of device (ECMO or Impella), pre-device cardiac arrest, center

^b^Model 2: adjusted by age, type of device (ECMO or Impella), shock to support time, center

^c^Model 3: adjusted by SOFA, SAPS 3, center

^d^There was no evidence of multicollinearity as assessed by the variance inflation test

^e^Overall lactate represents the mean serum lactate effect (1 h, 6 h, 12 h, 24 h) on 30-day mortality

^f^Clearance was calculated as the following: [(lactate at time point of interest – initial lactate)/initial lactate*100]

^g^All lactate clearance models were adjusted for initial lactate levels

**Fig. 1:**
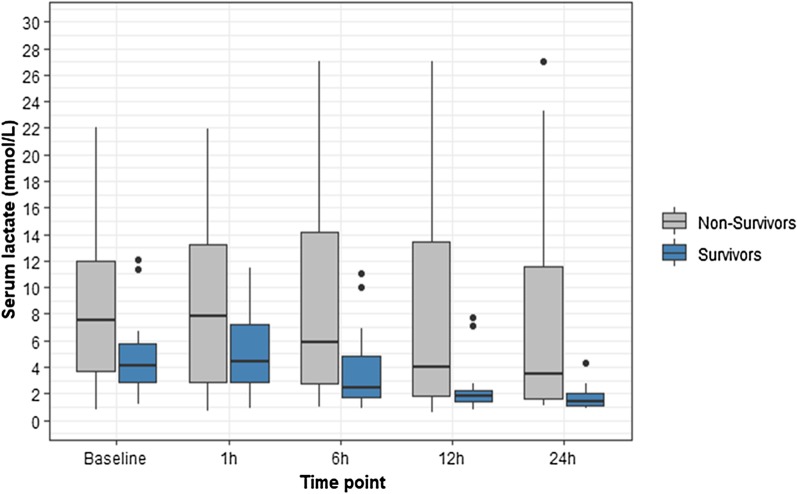
Differences in lactate levels at the time points of interest between survivors and non-survivors. Legend: The box plot inner horizontal lines indicate median; boxes, interquartile range (25th and 75th percentiles); vertical whiskers, 1.5 interquartile range beyond the 25th and 75th percentiles; and dots, more extreme values

Figure 1 depicts lactate levels and lactate clearance at baseline MCS, 1 h, 6 h, 12 h, and 24 h. Survivors showed a trend toward lower initial lactate levels in comparison to non-survivors (4.0 [2.6–6.3] mmol/L vs. 7.5 [2.8–12] mmol/L; *P* = 0.09). Lactate levels first rose in the first hour post-MCS placement, decreasing in subsequent hours. Significant differences between survivors and non-survivors was observed at 6 h (2.4 [1.7–6.2] mmol/L vs. 5.9 [2.6–15.0] mmol/L; *P* = 0.02), 12 h (1.8 [1.3–2.6] mmol/L vs. 4.0 [1.6–14.3] mmol/L; *P* = 0.02), and 24 h (1.3 [1.1–2.3] mmol/L vs. 3.5 [1.6–13.3] mmol/L; *P* = 0.001, Additional file 1: Table S5). Both survivors and non-survivors were able to clear lactate levels over time, but a statistically significant difference between them was seen only at 24 h (60.3% [42.5 to 72.8%] for survivors vs. 18.9% [− 50.0 to + 68.2%] for non-survivors, *P* = 0.04). After 24 h of MCS, 12 patients (28%) had higher lactate levels than at baseline. Decannulation (2 [17%] vs. 17 [55%], *P* = 0.02) and mortality rates (12 [100%] vs. 19 [61%], *P* = 0.01) differed significantly between patients who failed to clear lactate after 24 h versus those whose serum lactate improved during the first 24 h.

### Prediction of 30-day mortality

Lactate levels and lactate clearance showed an increase in area under the ROC curve (AUC) over time, with the greatest AUC at 24 h, but lactate levels showed better prognostic performance (Table 3).

**Table 3:** Receiver operating characteristic curves for prediction of 30-day mortality with lactate levels and lactate clearance

	AUC^a^	CI	*P*
Lactate, baseline	0.66	0.49–0.83	0.09
Lactate, 1 h	0.64	0.47–0.81	0.14
Lactate, 6 h	0.71	0.55–0.87	0.02
Lactate, 12 h	0.72	0.57–0.88	0.02
Lactate, 24 h	0.81	0.67–0.94	0.002
Clearance, 1 h	0.43	0.23–0.62	0.49
Clearance, 6 h	0.65	0.48–0.82	0.11
Clearance, 12 h	0.64	0.48–0.80	0.13
Clearance, 24 h	0.70	0.55–0.85	0.004

AUC, area under the curve; CI, confidence interval

^a^AUC: < 0.2 poor, 0.21–0.40 fair, 0.41–0.60 moderate, 0.61–0.80 good, 0.81–1.00 very good

**Fig. 2:**
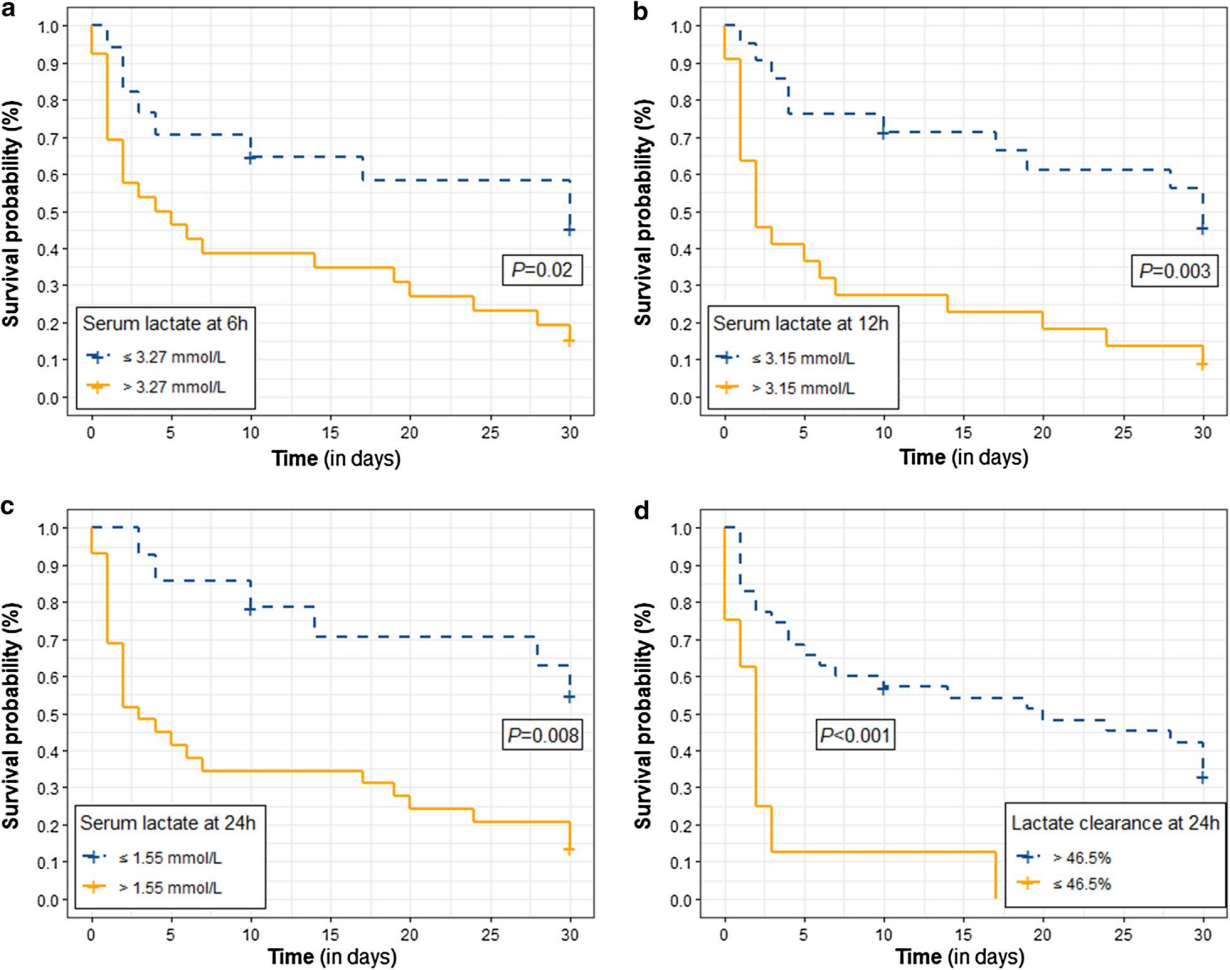
Kaplan–Meier curves of survival in groups stratified by lactate cutoff levels determined with Youden’s J statistic for area under the ROC curve. **a** Lactate at 6 h ≤ 3.27 mmol/L; **b** Lactate at 12 h ≤ 3.15 mmol/L; **c** Lactate at 24 h ≤ 1.55 mmol/L; **d** Lactate clearance at 24 h ≥ 46.5%

Youden’s J index was calculated only at time points of lactate levels or clearance which differed significantly between survivors and non-survivors. Serum lactate thresholds were identified at 6 h (3.27 mmol/L; S: 71%, PPV: 85%), at 12 h (3.15 mmol/L; S: 65%, PPV: 91%), and at 24 h (1.55 mmol/L: S: 81%, PPV 86%). The threshold for lactate clearance at 24 h was 46.5% (S: 74%, PPV: 66%). Kaplan–Meier survival curves for each of the lactate and clearance thresholds identified are shown in Fig. 2.

## Discussion

In this observational study of patients in cardiogenic shock treated with MCS, both serum lactate levels and lactate clearance were associated with 30-day mortality. In unadjusted and adjusted analyses, lactate levels at all time points and lactate clearance after 6 h were associated with mortality. Also, levels of serum lactate at 6 h, 12 h, 24 h and its clearance after 24 h were able to discriminate survivors from non-survivors in our cohort. These data demonstrate that lactate measurements may be of greater prognostic value than the amount of lactate cleared for a specific time point, although greater clearance was indeed associated with lower mortality. In addition, failure to clear baseline lactate levels after 24 h of MCS treatment was associated with 100% mortality. These findings may have practical implications regarding support strategies.

Serum lactate is a well-known outcome predictor in conditions associated with impaired tissue perfusion, such as cardiogenic shock [21, 22]. A cohort study of patients with cardiogenic shock who received percutaneous extracorporeal life support, mainly in the post-cardiopulmonary resuscitation setting, found that initial lactate level was able to discriminate survivors from non-survivors, after multivariable analysis [23]. These results, however, are in disagreement with previous studies [5, 8, 22]. The discrepancy in association of initial lactate levels with mortality may reflect time from insult to initiation of mechanical support, differences in shock etiology, and differences in comorbidity profile. In our cohort, unadjusted and adjusted analysis showed that the initial lactate level was associated with mortality. In this cohort, the median initial arterial lactate was 6.1 mmol/L (2.8–11.5 mmol/L). This value is supported by the literature as a threshold to trigger MCS, however, this cutoff is supported only by expert opinion. Previous cohorts of MCS patients have shown higher initial lactate levels, ranging from 7 to 14 mmol/L in the survival group than the one found in our study (4.0; 2.62 to 6.3), which may reflect a distinct population [5, 8, 14, 24]. Differences found in baseline lactate between survivors and non-survivors (4.0 vs. 7.5 mmol/L) in our study may be related to distinct cardiogenic shock etiologies and a higher burden of comorbidities such as hypertension and diabetes. However, survivors were more likely to have primary transplant graft failure and postcardiotomy syndrome, that are commonly associated with higher lactate levels due to the surgical insult. Despite these discrepancies, shock to support time, a possible cause for distinct initial lactate levels, and the cardiogenic shock risk scores SAVE and ENCOURAGE were similar between groups at baseline.

The improvement in lactate level after initiation of MCS reflects a hemodynamic response, and may have greater prognostic utility than initial lactate levels. However, there is no consensus regarding optimal clearance of lactate within time. Our study was the first to show that lactate early as 6 h can discriminate survivor from non-survivors. Also, different from previous studies that focused on specific cardiogenic shock etiologies, our results show that in several clinical scenarios the lactate levels in 6 h, 12 h, and 24 h can discriminate survivors from non-survivors. Indeed, lactate levels at 24 h was a better mortality predictor. This discriminatory power of the lactate over time, as shown in Table 3, is probably due to the improvement in tissue perfusion provided by clinical and MCS treatment. However, despite the improvement in arterial lactate over time, non-survivors still showed a higher level after 24 h [3.5 (1.6–13.3)] mmol/L in comparison to survivors 1.3 (1.1–2.3) mmol/L. These failure to decrease lactate levels in the same amount as the survivors may be related to other organ damage and not only by a direct effect of the lactate molecule. Differences in absolute lactate and in lactate clearance between survivors and non-survivors were found only after 12 h of support in a cohort of VA-ECMO with multiple etiologies [8]. Another recent study also highlighted differences in lactate levels in time, but failed to demonstrate any association of lactate clearance at 24 h with mortality [14]. However, it included a large number of postcardiotomy patients and did not include any with acute myocardial infarction, which may explain the different results found in our study. These data highlights that not only clearance, but also a lactate reduction to a normal level is associated with better outcomes.

Besides the lack of data in cardiogenic shock supported by MCS, the use of initial arterial lactate and its clearance have been used as an end-point for a better tissue perfusion in this scenario [25]. Initial studies on extracorporeal circulation demonstrated an association with inflammatory cytokines elevation due to the interaction of the blood with the device [26]. Similar concerns were raised on the use of ECMO for respiratory support in patients with COVID, where a cytokine storm have been reported [27]. Although the Impella catheter have a smaller contact surface in comparison to VA-ECMO, it produces a greater shear stress due to its axial pump design leading to hemolysis [9, 28, 29]. In addition, both devices were indicated for different cardiogenic etiologies as shown in Additional file 1: Table S1. In the attempt to evaluate the association of the device used (VA-ECMO or Impella CP) with the lactate levels, we included it in two out of three adjusted analysis, which showed similar results. These results highlight that, despite the possibility of some interaction with the device or the underlying condition, lactate level under MCS showed an expected kinetic and can be used as a prognostic biomarker.

Few interventions are able to change outcomes in cardiogenic shock. Refractory shock still carries high mortality rates despite MCS [30]. In this setting, lactate cutoff points can be a useful tool in the decision-making process. In an analysis of a cardiogenic shock registry, initial lactate greater than 4.0 mmol/L was associated with a sevenfold increase in mortality, and was proposed as a threshold for MCS escalation in the authors’ protocol [31]. Fux et al. [32] showed that an initial serum lactate greater than 15.0 mmol/L was associated with 100% mortality in the setting of refractory postcardiotomy shock. Fortunately, extremely high initial lactate is quite uncommon. In our cohort, the median initial lactate was 6.1 mmol/L. This median level is within the range where MCS may be considered (over 4.0 and below 11.0 mmol/L), i.e., not in the extremes where MCS may no longer be useful. Also, we showed that the 24 h lactate levels had clear prognostic significance; each mmol/L increase in lactate was associated with a 1.76-fold increase in mortality on adjusted analysis, rising up to 5.86-fold in a multivariable model. Youden’s J index identified clinically useful cutoff points: serum lactate levels below 3.27 mmol/L at 6 h, 3.15 mmol/L at 12 h, 1.55 mmol/L at 24 h, and a 24 h lactate clearance of 46.5% predicted better survival. Moreover, failure to improve lactate at 24 h was associated with only 17% decannulation, but 100% mortality, in this cohort. A similar trend was seen in a recent cohort of cardiogenic shock patients treated with IABP, where a lactate of 3.1 mmol at 8 h was the best predictor [13]. These data have the potential to affect bedside clinical decisions. Both VA-ECMO and Impella CP^®^ are costly interventions, which require rational use to be cost-effective [33]. Not only are the initial costs of the devices themselves high, but their use may entail additional procedures that are needed to support a critical patient, such as continuous hemodialysis, antibiotic therapy, mechanical ventilation, and prolonged ICU stay. If failure to improve lactate level after 24 h have such a clinical impact, and it is validated by further studies, it should be incorporated to the clinical decision process.

Rational decisions regarding implantation and weaning of MCS devices are paramount determinants of the treatment success rates and financial sustainability of critical care programs. In this scenario, it is reasonable to use lactate as a biomarker to assess disease severity. Lactate has all the characteristics of a good biomarker: it is widely available as a point-of-care test, inexpensive, and noninvasively measured. However, it should always be used in light of the clinical data available, never as a single variable.

### Study strengths and limitations

The strengths of this study rely on its multicentric design and the fact that all centers had uniform training on the use of MCS devices. Moreover, to the best of our knowledge, this is the first study to measure lactate kinetics using both VA-ECMO and Impella CP^®^. Data on the use of MCS devices in low- and middle-income countries are scarce, and their cost-effectiveness in this scenario is yet to be established. Our study design has several limitations. This is a non-planned retrospective analysis from a cohort of MCS in cardiogenic shock. The four centers enrolled are not considered high volume for MCS in cardiogenic shock and it may have an impact in the indication and management of the cases. Due to the small cohort size, lactate kinetics could not be analyzed separately for each shock etiology or device. The choice of VA-ECMO or Impella CP^®^ implantation followed local protocols, which took into account several clinical characteristics in the decision process other than initial lactate levels alone. Center-specific protocols may have differed regarding vasopressor support, fluid resuscitation, and other clinical management decisions that can influence lactate levels and the patient outcomes. Higher lactate levels in the non-survivor group may have reflected underlying disease severity rather than failure to clear lactate while on MCS. Finally, the high mortality rate seen in our cohort also may have been attributable to illness severity, which may have affected the results of analysis. More studies are warranted to improve our understanding of lactate kinetics on cardiogenic shock and its role as a prognostic marker.

## Conclusion

Arterial lactate is a biomarker currently used to assess prognosis in patients with cardiogenic shock treated with MCS devices (ECMO and Impella CP^®^). The evaluation of lactate kinetics in this scenario showed a stronger relationship with 30-day mortality with the absolute lactate levels after 6 h of support in relation to lactate clearance in 24 h. The best prognostic ability was associated with absolute lactate levels at 24 h.

## Supplementary information

**Additional file 1**. Supplementary material.

## Data Availability

The datasets of this study are not publicly available due to bylaws of the Brazilian Ministry of Health, but may be available from the corresponding author on reasonable request and legal permission.

## Abbreviations

AUC: Area under the ROC curve
CI: Confidence interval
COPD: Chronic obstructive pulmonary disease
ENCOURAGE: Prediction of cardiogenic shock outcome for acute myocardial infarction patients salvaged by VA-ECMO
IQR: Interquartile range
MCS: Mechanical circulatory support
OR: Odds ratio
PPV: Positive predictive value
ROC: Receiver operating characteristic
SAPS3: Simplified Acute Physiology Score III
SAVE: Survival After Veno-arterial ECMO Score
SOFA: Sequential Organ Failure Assessment Score
VA-ECMO: Veno-arterial extracorporeal membrane oxygenation
