# Supplementary material for: Association between serum lactate levels and mortality in patients with cardiogenic shock receiving mechanical circulatory support: a multicenter retrospective cohort study
Source: BMC Cardiovasc Disord. 2020 Nov 24;20:496. doi: 10.1186/s12872-020-01785-7 (PMC7687839; doi:10.1186/s12872-020-01785-7)
Supplement: Supplementary file 1 — Additional file 1. Supplementary material. [file 12872_2020_1785_MOESM1_ESM.docx]

**SUPPLEMENTARY TABLES**

**Supplementary Table 1.** Cardiogenic shock etiology and device used at each center enrolled.

|  | **Center 1**  **(n=4)** | **Center 2**  **(n=24)** | **Center 3**  **(n=8)** | **Center 4**  **(n=7)** | **VA-ECMO**  **(n=30)** | **Impella CP**^®^  **(n=13)** |
| --- | --- | --- | --- | --- | --- | --- |
| Acute myocardial infarction (%) | 4 (21%) | 11 (58%) | 4 (21%) | - | 100 (53%) | 9 (47%) |
| Acute decompensation of chronic heart failure (*%*) | - | 4 (40%) | 2 (20%) | 4 (40%) | 8 (80%) | 2 (20%) |
| Primary transplant graft failure (%) | - | 3 (75%) | - | 1 (25%) | 4 (100%) | - |
| Cardiac arrest (%) | - | 3 (75%) | - | 1 (25%) | 2 (50%) | 2 (50%) |
| Postcardiotomy syndrome (%) | - | 1 (33%) | 1 (33%) | 1 (33%) | 3 (100%) | - |
| Pulmonary thromboembolism (%) | - | 1 (50%) | 1 (50%) | - | 2 (100%) | - |
| Myocarditis (%) | - | 1 (100%) | - | - | 1 (100%) | - |
| VA-ECMO (%) | 2 (7%) | 16 (53%) | 5 (17%) | 7 (23%) | - | - |
| Impella CP^®^ (%) | 2 (15%) | 8 (62%) | 3 (23%) | - | - | - |

VA-ECMO, veno-arterial extracorporeal membrane oxygenation

**Supplementary Table 2**. Unadjusted analysis of age, device, pre-device cardiac arrest, center, Simplified Acute Physiology Score III (SAPS3) and Sequential Organ Failure Assessment Score (SOFA), door-to-support time as mortality predictors in cardiogenic shock patients receiving mechanical circulatory support.

|  | **Unadjusted** | | |
| --- | --- | --- | --- |
|  | **OR** | **95% CI** | ***P*** |
| Age (years) | 1.00 | 0.96-1.05 | 0.72 |
| Device type^a^ | 2.05 | 0.50-8.34 | 0.31 |
| Pre-device cardiac arrest | 2.87 | 0.71-11.52 | 0.13 |
| Center 1 | 0.16 | 0.00-2.98 | 0.22 |
| Center 2 | 0.40 | 0.04-4.00 | 0.43 |
| Center 3 | 0.50 | 0.03-7.10 | 0.60 |
| Center 4 | Reference | 0.02-6.06 | 0.52 |
| SAPS 3 | 1.09 | 1.02-1.15 | 0.003 |
| SOFA | 1.07 | 0.89-1.29 | 0.44 |
| Shock to support time (h) | 0.90 | 0.19-4.33 | 0.90 |

CI, confidence interval; OR, odds ratio; SAPS 3, Simplified Acute Physiology Score III; SOFA, Sequential Organ Failure Assessment score

^a^Device type: ECMO or Impella.

**Supplementary Table 3**. Table S2: Sensitivity analysis of serum lactate and lactate clearance for missing inputs.

|  | **Unadjusted** | | |
| --- | --- | --- | --- |
|  | **OR** | **95% CI** | ***P*** |
| Lactate, baseline (mmol/L) | 1.15 | 1.00-1.38 | 0.44 |
| Lactate, 1h (mmol/L) | 1.13 | 0.98-1.35 | 0.06 |
| Lactate, 6h (mmol/L) | 1.14 | 1.00-1.41 | 0.03 |
| Lactate, 12h (mmol/L) | 1.26 | 1.04-1.76 | 0.03 |
| Lactate, 24h (mmol/L) | 1.80 | 1.10-4.63 | 0.006 |
| Clearance, 1h (%)^a^ | 0.99 | 0.98-1.01 | 0.86 |
| Clearance, 6h (%)^a^ | 0.98 | 0.97-1.00 | 0.07 |
| Clearance, 12h (%)^a^ | 0.98 | 0.96-1.00 | 0.04 |
| Clearance, 24h (%)^a^ | 0.98 | 0.96-0.99 | 0.01 |

CI, confidence interval; OR, odds ratio;

^a^ Clearance was calculated as the following: [(lactate at time point of interest – initial lactate)/initial lactate*100]

**Supplementary Table 4**. Table S2: Sensitivity analysis of serum lactate and lactate clearance for cardiogenic shock etiology.

|  | **Unadjusted** | | |
| --- | --- | --- | --- |
|  | **OR** | **95% CI** | ***P*** |
| Lactate, baseline (mmol/L) | 1.02 | 1.00-1.04 | 0.02 |
| Lactate, 1h (mmol/L) | 1.02 | 1.00-1.04 | 0.05 |
| Lactate, 6h (mmol/L) | 1.02 | 1.00-1.04 | 0.02 |
| Lactate, 12h (mmol/L) | 1.02 | 1.00-1.04 | 0.02 |
| Lactate, 24h (mmol/L) | 1.03 | 1.01-1.05 | 0.003 |
| Clearance, 1h (%)^a^ | 1.02 | 0.80-1.30 | 0.85 |
| Clearance, 6h (%)^a^ | 0.90 | 0.81-1.01 | 0.08 |
| Clearance, 12h (%)^a^ | 0.95 | 0.91-0.98 | 0.01 |
| Clearance, 24h (%)^a^ | 0.95 | 0.92-0.99 | 0.01 |

CI, confidence interval; OR, odds ratio;

^a^ Clearance was calculated as the following: [(lactate at time point of interest – initial lactate)/initial lactate*100]

**Supplementary Table 5**. Lactate levels and clearance in several time point between survivors and non-survivors.

|  | **Survivors** | | **Non-survivors** | |  |
| --- | --- | --- | --- | --- | --- |
|  | **Median** | **IQR** | **Median** | **IQR** | ***P*** |
| Lactate, baseline (mmol/L) | 4.0 | 2.6 to 6.3 | 7.5 | 1.8 to 12.0 | 0.09 |
| Lactate, 1h (mmol/L) | 4.4 | 2.0 to 8.3 | 7.8 | 2.5 to 14.4 | 0.14 |
| Lactate, 6h (mmol/L) | 2.4 | 1.7 to 6.2 | 5.9 | 2.6 to 15.0 | 0.02 |
| Lactate, 12h (mmol/L) | 1.8 | 1.3 to 7.5 | 4.0 | 1.5 to 14.3 | 0.02 |
| Lactate, 24h (mmol/L) | 1.3 | 1.1 to 2.2 | 3.5 | 1.6 to 13.3 | 0.001 |
| Clearance, 1h (%)^a^ | -12.1 | -37.9 to 16.3 | 0 | -25.4 to 15.8 | 0.51 |
| Clearance, 6h (%)^a^ | 26.5 | -9.1 to 48.2 | -10.0 | -47.0 to 45.3 | 0.12 |
| Clearance, 12h (%)^a^ | 48.8 | 32.3 to 64.8 | 24.5 | -50.0 to 60.9 | 0.13 |
| Clearance, 24h (%)^a^ | 60.3 | 42.5 to 72.8 | 18.9 | -50.0 to 68.2 | 0.04 |
| IQR, interquartile range (p25-p75).  ^a^ Clearance was calculated as the following: [(lactate at time point of interest – initial lactate)/initial lactate*100] | | | | | |
